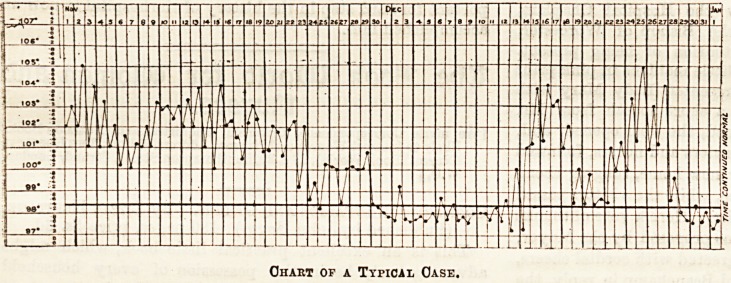# The Hospital. Nursing Mirror

**Published:** 1898-04-23

**Authors:** 


### "The Hospital" Nursing Mirror.

Being the Nursing Section of "The Hospital."

[Contributions for this Section of "The Hospital" should be addressed to the Editor, The Hospital, 28 & 29, Southampton Street, Strand, London, W.C., and should have the word "Nursing" plainly written in left-hand top corner of the envelope.]

### News from the Nursing World.

### AT THE LONDON HOSPITAL.

The wards at the London Hospital boast many additions which must rejoice the hearts alike of sisters and nurses. The white tiled tables, new lockers here and there, the splendid beds which are found in every ward, sundry new armchairs, the floors covered with linoleum, and many another improvement are pleasant to see. The nurses are to be congratulated, too, on the neat and pretty Sister Dora caps, without strings, which have been theirs since the beginning of the year. They are a great improvement on the old caps which were wont to take a certain variety of shapes on the heads of their different owners, with a result not altogether uniform. There is perhaps no cap more becoming and nurselike than the one now adopted. The Sisters' caps remain as of yore.

### THE HOME FOR INCURABLE CHILDREN, MAIDA VALE.

All who know anything of this admirable home will hear with sincere regret that Miss Coleman, after fourteen years of most devoted work, is soon giving up her post as matron. Greatly will she be missed, both by the committee, by her nurses, and by the children over whom she has watched so carefully all these years.

### A NEW NURSES' HOME.

The foundation stone of the "Victoria Nurses' Home" was laid at Solihull the other day by Miss Chattock, the lady by whom the entire cost of the building is being defrayed. The site for the Home is given by Mr. and Mrs. Herbert Wright, and these generous gifts have made it possible for the amount collected in connection with the Jubilee Commemoration Fund to be formed into the nucleus of an endowment fund. For the past two years Miss Chattock has herself maintained a nurse in Solihull, and when the question of a Jubilee memorial was mooted offered to provide a building if the money to support a permanent nurse could be got together. Some £700 has been contributed, and a further sum of £49 was collected on the day of the stone laying. It is hoped that a sum of £3,000 will ultimately be placed to the credit of the endowment fund.

### DISTURBANCES AT LLANELLY.

It appears on inquiry respecting the resignation of the matron and staff of the Llanelly Hospital that the absence of definite regulations has been the cause of the disturbance. The matron probably proceeded to effect reforms when she had only been resident in the hospital for a very short time, and took informal instructions from some of the committee in the absence of definite rules. It is a pity that matters should have come to the pass they have, but doubtless the committee will now see the advisability of instituting proper regulations at their institution. We learn that the committee of management have decided to close the institution at the expiration of the services of the nurses, and to discharge all the patients and restaff the hospital as speedily as possible.

### THE MIDWIVES' INSTITUTE.

The course of instruction for the July L.O.S. examination is announced to begin at the Midwives' Institute, 12, Buckingham Street, on April 28th, and that for the October examination on August 23rd. The lectures are given, as usual, by Dr. F. R. Humphreys, and take place on Monday and Thursday afternoons. They are supplemented by nine lectures on monthly nursing by Mrs. Hewer. Classes by an experienced midwife are also held for the benefit of those pupils who may desire some further help and instruction. The fee for the course of 27 lectures is £3 3s., for a single lecture 2s. 6d., and for nine coaching classes 15s. Arrangements have been made with some of the midwife members to reserve vacancies for pupils recommended through the Institute, and those who wish to avail themselves of this opportunity are asked to communicate with the secretary without delay.

### THE NEW HOME AT WORCESTER.

It was a real pleasure to be present at the opening of the new nurses' home at the Worcester General Infirmary on Tuesday last. Hospitals have very different atmospheres; at some a feeling of good-fellowship is in the very air, while at others there is a moral tension which cannot fail to make itself felt. At Worcester a thoroughly home-like feeling pervades everything. Miss Herbert keeps alive the courteous and kindly spirit of her Nightingale training school, and it was very clear to an observer that the "home" will be so not in name only. It was a pretty thought to arrange for the nurses to enter their own new home before anyone else, and a detail not without significance. At some hospitals the board-room is well and handsomely furnished, while the nurses' floors are bare and their chairs hard. At Worcester a shabby carpet does duty for the governors, while every effort is made to ensure that the nurses shall have those comforts which will send them back to their work in the wards rested and refreshed. When this is the spirit abroad in a hospital, how easy it is for all to work together for the good of the patients.

### SWEATING AT COVENTRY INFIRMARY.

Are there no women on the Coventry Board of Guardians to protest against the treatment which appears to be there considered good enough for an unfortunate night nurse? At a recent meeting of this board a letter was read from Nurse Turner stating that she was off duty but one night a month in lieu of the half-day fortnightly granted to the rest of the staff. She asked for a half-day a month, and added, "I have been in your service for the past five months, during which period I have only had one night off duty. I find mine to be a most trying post, for it is impossible to sleep through the noise and bustle on all sides of my bedroom; I could count the hours that I sleep through each week." There are no words strong enough to condemn the inhumanity which not only expects a woman to continue night nursing for five months with only one night off duty, but allows her to occupy a sleeping room which can be one only in name. The letter was referred to the Infirmary Committee, one of the guardians remarking that "it showed the urgent necessity of pushing on the nurses' new department." We agree with him. While such things are being daily reported from provincial unions is there any wonder that guardians find it next to impossible to secure the services of good nurses?

### A HASTY JUDGMENT.

The writer in a recent *Lancet* who, in condemning institutions "run" by medical men, mentioned in this undesirable connection St. Catherine's Home for Incurables, Bradford, must surely have done so in the absence of any personal knowledge of this excellent institution and its good work. The home, which has been in existence some five years, was not founded by a medical man at all, but by a few ladies whose wish was to provide a refuge for dying and hopeless cases such as could not be retained in general hospitals. Neither has the medical profession been represented on the executive body, for the Home is and has always been under the management of a committee of ladies, Mrs. Bottomley being its president, and Mrs. Rabagliati the hon. secretary. A preference is given to cases of cancer, but no incurable disease is refused admission except for want of room. All who know St. Catherine's rejoice that the generosity of a friend has made it possible to extend its work. In June the Home will be moved to larger quarters.

### A SUGGESTION.

The annual meeting of the Salisbury Institution for Trained Nurses was reported in the *Salisbury Journal* the other day, and in the same paper appeared a letter from Mr. Weigall containing a suggestion which deserves careful consideration, both on its merits and as coming from one whose interest and experience in all matters charitable is well-known in Salisbury. The nurses belonging to the Institution, or Diocesan Home, are trained at the Salisbury Infirmary, but the two institutions are wholly distinct in management, and have no other connection with each other. Mr. Weigall asks whether it might not be in the interests of the city if the one could now be merged in the other, the new Nursing Home which is to be added to the Infirmary being made large enough to accommodate the institution nurses, the management of the latter being placed under the control of the Infirmary Committee, and the matron of the infirmary undertaking their superintendence. This would mean economy in working expenses and upkeep generally, with other advantages too many to mention here. There are two objections to be faced. One is that the change might lead to a loss of subscriptions to the Nursing Institution, which at present undertakes a certain number of gratuitous cases; the other that the standard of nursing amongst the private nurses must, if the amalgamation took place, then rise and fall with that at the infirmary, *i.e.*, should at any time the nursing at the infirmary be vested in the hands of a less competent matron, the private nursing institution would also suffer. On the other hand there can be no doubt that, given a competent matron, her intimate knowledge of the nurses trained under her own eye in the wards will enable her to make the best possible selection of those fitted for private work. Of course there are reasons against as well as for such a change, but on the whole the advantages would seem to outweigh the disadvantages, and the committee of the Diocesan Home should think well before they lose the present opportunity.

### LONDON SCHOOL OF MEDICINE FOR WOMEN.

It is announced that the Princess of Wales has consented to open the new laboratories at the London School of Medicine for Women in Handel Street, Brunswick Square, in July. The Prince of Wales will also be present.

### NURSES' HOME, NEWCASTLE.

A satisfactory report was laid before the annual meeting held at the Nurses' Home, Granville Road, Newcastle, last month. Five hundred and eighty-eight cases were nursed during the year, the staff numbering some 70 nurses, amongst whom the committee had been able to distribute a sum of £325 as bonus money. The committee place on record in their report their recognition of the "able and faithful assistance received from both the matron and assistant matron."

### A PLEASANT ENTERTAINMENT.

A bright and pleasant entertainment, under the direction of the Secretary, was held in the City of London Hospital for Diseases of the Chest, Victoria Park, E., last week, for the amusement of the patients. The programme comprised items of a most amusing variety, including a musical sketch. Many ladies and gentlemen kindly assisted, and several members of the committee and staff were present. Mr. Charles Thomerson gave a most clever imitation upon the piano of many musical instruments, his duet, "Twins," with Mr. Woods, being most enthusiastically received.

### IN MEMORIAM.

The late Countess of Lathom, whose death took place recently under such tragic circumstances, was a good friend to all charitable undertakings in South-West Lancashire, and it is appropriate and fitting that her memory should be perpetuated by some such scheme as the one now under consideration. This is to found an endowment for the maintenance of a district nurse to work in the districts of Lathom and Skelmersdale. A sum of £3,000 is required for this purpose, and it is suggested that the nurse shall live in the home attached to the Cottage Hospital at Ormskirk.

### SHORT ITEMS.

The Carlisle Nursing Association reports a falling off in subscriptions during the past year, a very regrettable fact seeing the good work of which it is also able to give account. The appeal for more help should meet with prompt response.—The annual meeting of the Hartlepool Nursing Guild was held the other day. The report showed a satisfactory balance in the bank to the credit of the association.—A nursing association is about to be started at Brecon to continue the work of district nursing on more definite lines than has as yet been attempted in the town.—A sum of £36 12s. 4d. has been handed over to Major-General Lee, the treasurer of the Barry Nursing Association, as the result of the first day's collection made on its behalf by the Barry Trades' Council.—At the annual meeting of the Bishop Auckland District Nursing Association a vote of thanks was passed to the nurses for their services during the past year and satisfaction was expressed at the work done.

### Antiseptics for Nurses.

By a Medical Woman.

### VIII.—CLEANSING OF THE SKIN—DRESSINGS.

The cleansing of the skin before an operation is very necessary, and it is extremely important that this should be done thoroughly and intelligently. Germs of all sorts abound on the skin, for it combines all those conditions which are most favourable to their growth, viz.: (1) Moisture, which is supplied by the secretions of the glands of the skin, or of the mucous membrane; (2) equable temperature; whilst (3) the dead cells on the surface of the skin, as well as other substances, supply the necessary nourishment. It is said that each change of occupation leaves behind its mark in bacteria, even with common cleanliness, so constantly are we coming into contact with substances that harbour bacteria, and so readily do these attach themselves to the skin. Bacteria adhere to and increase in some parts of the body more quickly than in others; these are especially parts covered with hair, and where the secretion of perspiration is profuse. The cavity of the mouth, the whole alimentary track, and the upper part of the respiratory track harbour immense numbers of bacteria. From this it will be evident that "surgical cleanliness" is not the simple matter some people seem to think.

The best substance for the preliminary cleansing is soap—any form of the better kinds of soap may be used—the essential point being that the soap has been prepared by the hot process, that is, by boiling, since thus all germs are destroyed. The common hard soap is not desirable, since it is prepared by the so-called "cold process," and, consequently, the bacteria that abound in animal fats are not destroyed. The hands and arms, as far as the elbow, at all events, must be thoroughly scrubbed with a brush; and plenty of soap and water, as hot as can be comfortably borne, for at least five minutes, special attention being paid to the nails and the spaces under the nails. The hands should be rinsed in fresh hot water, and then should be dipped into a basin of methylated spirit, and well rinsed in this, and finally soaked for a minute in perchloride of mercury, 1 in 1,000. This process must be conscientiously carried out by the nurse, and no part of it omitted. A nurse cannot be too deeply impressed with the difficulty of thoroughly cleansing her hands and getting rid of the bacteria which lie embedded in the greasy secretions of the skin, in every crack and crevice. Formerly, that is, about ten years ago, at the beginning of the antiseptic era, when everyone had the most unlimited faith in carbolic, the process of disinfecting the hands was of the very simplest, and consisted in merely dipping the hands into carbolic for one or two minutes, and then it was thought that disinfection was complete, and washing previously with soap and water was thought quite superfluous. This dipping even in strong carbolic is now known to be absolutely inefficient, because there are no bacteria more difficult to kill than those of the skin, surrounded as they are by greasy material, and even strong antiseptics have no effect on this oily protective covering, whilst corrosive sublimate runs off greasy skin in drops, not even moistening it, and the germs remain quite untouched. Thus, the most important elements in the disinfection of the skin is to dissolve off the dirt in which organisms can lodge by the use of as much hot water and soap as possible, supplemented by alcohol or ether. A number of disinfectants are recommended from time to time as the best and most active for this purpose, but chemical disinfectants are of very minor importance, and many authorities discard antiseptic agents for disinfecting the skin altogether and practise only the most minute and careful washing, and with very good results. The disinfection of the skin is not a question of using any particular disinfectant, but of the most scrupulous care and the greatest intelligence.

Of course, a patient's skin over and around the area of operation should be prepared with equal if not greater care. After the cleansing process already described has been carried out, the part should be covered with a double piece of lint, extending a good distance beyond the probable seat of operation, and covered with mackintosh or protective reaching well beyond the edges of the lint, and the whole kept in place with a bandage or binder and only removed in the operating-room. If the patient's skin is very dirty, and the dirt is ingrained or the skin peels off, it should be well rubbed with ether before it is scrubbed with soap and water.

Care must be taken that the brush used for these disinfecting processes is itself aseptic. Any brush taken up casually, and that has already been used for a variety of purposes, will not do, as it is itself swarming with germs. A special brush should be reserved exclusively for the purpose, and when not in use it should be kept in a jar containing perchloride of mercury, 1 in 5,000, changed daily. The brush must, of course, have been thoroughly cleansed previously. Some authorities recommend filling the intervals between the nail and the fingers, as well as the grooves round the nails, with a firm aseptic paste, the one proposed consisting of carbolic acid and camphor, so as to prevent any danger of contagion, but it has not been possible up to now to make a paste which will not crack and fall out. Perhaps, collodion is, on the whole, the most satisfactory for the purpose.

If it falls to the lot of a nurse to hand the instruments during an operation, or in any way to directly help the surgeon as his assistant, she must bear in mind that, having once thoroughly disinfected her hands, she must not touch other things. If a table is not in the most convenient position, for instance, or some help is required in managing the patient, she should not be the one to do these things if there is anyone else available. If there is not, then she must on no account forget to disinfect her hands again after contact with anything that has not been rendered aseptic.

It is always difficult to secure absolutely aseptic dressings, but the nurse must do her best to prevent any contamination. It is advisable for her, after taking all precautions, to arrange the dressings that will be required for an operation in a closely-fitting tin box, which shall be taken into the operating-room, and when the dressings are required she should hand the box to the surgeon and not handle them herself, unless she has taken the precaution to make her hands aseptic immediately before doing so.

### Where to Go.

A Floral Bicycle Gymkhana, to be followed by a Cinderella Dance, will be given at the Portman-rooms on Thursday, May 5th, by Mr. A. Wheaton and his pupils in aid of the Cottage Hospital, Southwold.

Royal British Nurses' Association.—The last sessional lecture of the season will be given by Miss Georgina Scott (late matron of the Sussex County Hospital) on Friday, April 22nd, at eight p.m., at 11, Chandos Street, Cavendish Square, on "The Recreations of Working Women."

Zenana Bible and Medical Mission.—Annual meeting, New Hall, 26, George Street, Hanover Square, Friday, 29th, at three p.m. At an evening meeting at the same date and place limelight views will be exhibited.

### Novelties for Nurses.

### NEW LETTER-CARDS.

The Sovran Card Company have produced a useful letter card which is especially adapted for enclosing circulars, and a reply post-card of various forms and for various purposes. Samples can be sent from the company, 29, King Street, Covent Garden, W.C.

### Nursing in Paris Hospitals.

### B.—THE TRAINING OF NURSES.

### I.—Institution of Schools.

Even the opponents of the introduction of lay nurses into the Paris hospitals have to confess that the dispossessed sisters have themselves largely to blame for the change. The march of modern science, and of medical and surgical science above all, has required complete changes in hospital matters, and a great amount of technical knowledge in nurses, as well as in surgeons. Moreover, it has been requisite for nurses to be constantly learning new improvements in their work. Unfortunately, the nursing sisters, though faithful, were by nature conservative, and made no efforts themselves to keep in touch with the new developments in hospital work. There is no evidence to show, however, that the sisters would not have consented to adapt themselves to the new conditions, and to receive the necessary instruction.

The first public move for the establishment of schools for lay nurses was made in 1875. It is worth noticing, however, that an administrative essay was made as far back as 1853 in that historic cradle of so many hospitaliary reforms, La Salpétrière. In that great school of modern treatment for the insane, the idiot, and other classes of the mentally afflicted, a school for lay nurses was started, but as at that time almost all the hospitals were under the care of the sisters probably the limited field of action made the affair premature. Twenty years later, however, when the third Republic had replaced the Empire, and the Paris Municipal Council was beginning its aggressive career. a bosom friend of Dr. Bourneville, M. Talendier, being a member of the parliament of the capital city, to break the ice for laicisation, succeeded in making the affair a public question. In 1875 the motion in the Municipal Council made by M. Talendier, at the instigation of Dr. Bourneville, was deferred until November 20th, 1877, when Dr. Bourneville, having himself become a member of the Council, succeeded in carrying an order for the inauguration of the first schools, two in number, the first being appropriately at the scene of the old attempt—La Salpétrière—and the other at Bicêtre. These were opened respectively on April 1st, 1878, and on May 24th, 1878. A third school was established at La Pitié on May 26th, 1880. Finally, a fourth school was established at the Lariboisière Hospital, being unofficially organised December 11th, 1894, and officially recognised by M. Peyron April 6th, 1895. The Lariboisière school was started to relieve the crowded state of the schoolrooms at La Salpétrière and La Pitié, and selected as a centre for the northern group of hospitals—St. Louis, the Maison de Santé, and Bichat.

Before the establishment of the Lariboisière school the budget for the three others amounted to 16,100 frs., including 9,500 frs. as teachers' salaries, 3,500 frs. printing, books, &c., and 3,100 frs. for prizes. Besides this, in 1879 an annual subvention of 20,000 frs. for scholarships was appropriated by the city, but in the course of time these occasioned considerable jealousy, and the appropriation was converted to other purposes, and in 1897 an order was made for the diversion of the fund after completion of the terms of the existing holders of the scholarships. The total appropriation of the city of Paris for the nursing schools last year was 19,400 frs., the items not being stated. Besides this the still remaining *bourses* had 6,000 frs. alloted to them, while a special grant of 4,700 frs. was given to the practical courses for nurses, making 30,100 frs. in all. This item for practical instruction is presumably for the special courses at hospitals outside the regular schools, to which I shall allude in my account of "Practical Instruction."

The number of schools is not likely to be increased, although the attendance of nurses from other hospitals is, of course, made at considerable inconvenience, especially as such attendance is deducted from their scanty hours of rest. In some cases, where the hospitals are too distant from the nearest school, the director furnishes a conveyance to the students. One might imagine from this that efforts would be made to inaugurate a school in each hospital. Far from this; the promoters of the schools evidently fear for the existence of the present ones. Dr. Bourneville, the director of the schools, their founder and apostle, is continually exhorting the certificated nurses to get transferred to hospitals without schools that the enemy (*i.e.*, the opponents of laicisation) shall not criticise the schools as inordinately expensive, should the number of pupils become small because many of the nurses have got their diplomas.

Another pet project of Dr. Bourneville's is to diminish the number of the present class of nursing schools by turning the one at La Pitié into an "Ecole de perfectionnements," a project I shall have occasion to briefly allude to in a subsequent article. It is very doubtful, however, whether the administration will sanction much more additional expense in this direction. Thus the primary schools, which I also will allude to later, are clearly an extra burden occasioned by laicisation. The religious organisations either had recruits of sufficient elementary education, or bore themselves the expense of inculcating such education. On the other hand, the laicisers can contend that the additional expense of the professional schools is justified by their supplying a class of nurses with expert modern qualifications not possessed by the sisters. I cannot say that I have found all the hospital directors willing to back up this contention by their experience. In fact, one of them told me in confidence that he did not consider the new-fangled certificate as worth a straw (I am sorry to say he used a much stronger expression) as evidence of the value of the nurse.

So long as the present director is at the head of the Assistance Publique there is not likely to be any lessening to the support of the schools, nor any less encouragement given to the holders of certificates, by promotions and recommendations to other employments, such as country hospitals, private nursing, dispensaries, ambulances, &c. In this M. Peyron faithfully carries out the dominant idea in the Municipal Council, his indirect employer; for the Council, as the father of the schools, is not disposed to abandon its child. Perhaps the minority element, which supports the system of sisters, which has succeeded in retaining them in the two largest hospitals, and may possibly succeed in the project of new hospitals under the sisters, will take a leaf out of their opponents' book and ask for the creation of a special school for the religious nurses.

Edmund R. Spearman.

### The Nursing of Mediterranean Fever.

THERE are one or two points connected with Mediterranean fever that may prove of interest, from a nursing point of view, to those who have not come across cases of the disease.

The term "Mediterranean" is now generally used to designate the relapsing fever so prevalent at places on the shores of the inland sea, it being recognised that the disease is one and the same thing whether it hails from the Rock or the islands of Malta.

Few British residents at either place escape without a touch, at least, of this most trying complaint, though some may live a year or two exposed to its influence, with seeming immunity, and go back to England congratulating themselves on getting off so easily, only to smile on the other side of their faces, when, as is often the case, they are seized with a severe attack, probably lasting for months.

CHART OF A TYPICAL CASE.

The first symptoms of Mediterranean fever are not unlike the initial signs of most acute febrile diseases. They consist of severe headache, great lassitude and thirst, furred tongue, pains in the limbs, loss of appetite, sometimes accompanied by diarrhœa. The incubation period seems to be uncertain, some patients being suddenly seized with acute symptoms, while others will go on for days struggling against the increasing weakness and weariness till forced to give in.

The aspect of a Mediterranean case is remarkably like that of typhoid, except that spots are absent. The stools are identical in colour and consistence, though as a rule the diarrhœa is not so profuse. This similarity has made the new serum reaction test of great value, and through its agency it is said that typhoid, or Mediterranean fever, can be at once diagnosed. This, from a nursing point of view, is a decided gain, as cases of the latter can have *rather* a more generous and varied diet, and need not be kept in the strictly recumbent position so necessary in typhoid. The fever usually runs such a long and tedious course, that any little comfort or change the sufferers can have tends to make their illness more bearable.

It is no uncommon thing for a patient to remain in hospital for three or four months, going through periods of high fever, sometimes reaching 107 deg., followed by a week or two of normal and subnormal records, during which he is generally able to get up for part of the day. This happy condition soon changes, and for no apparent reason the temperature will dash up again, and another wave of fever sets in, frequently associated with rheumatic pains in the joints. This latter is one of the most distressing complications of Mediterranean fever, and the last to disappear. Inflamed glands in the axilla, or groin, and crops of boils in various parts of the body are not uncommon accompaniments, and bed sores have to be strictly guarded against.

Perspiration is excessive, and unless the backs of patients receive daily attention, a curious kind of sore is very quickly formed, not at all like the ordinary bed sore, but a long narrow crack, which if not attended to at once rapidly deepens. These do not occur on the seat of pressure, and seem to be caused by the irritation of perspiration. Should one develop it must be dressed twice a day, or oftener if the dressing gets soiled, with resin ointment spread on lint. This is the only application I have found of the slightest use. A fever-crack once formed never heals until the temperature comes down, but with care can be prevented from increasing. The mouth, teeth, and tongue are usually extremely foul, and require constantly cleaning with lemon, glycerine and borax, or some other similar wash. Delirium is frequently present, but is not often of so violent a nature as in many other acute diseases. After a long course of high fever the patient feels so thoroughly weak and languid that he usually acquiesces in any treatment considered necessary, and resigns himself to lying in bed week after week, and taking the same monotonous diet. Absolute rest, a very light fluid diet, generally supplemented by stimulants, are usually ordered, cold sponging and ice baths being also used to reduce temperature. One highly successful method of bathing is to place a sheet mackintosh under the patient in bed; then allow cold water to flow over his body from an india-rubber tube leading from a vessel placed on a high stool or table at the head of the bed.

The appended chart is a typical one of a moderately long attack.

### Appointments.

### MATRONS.

FOUNTAIN FEVER HOSPITAL, LOWER TOOTING.—Miss E. J. West has been appointed Assistant Matron to this hospital. Miss West was trained at St. Bartholomew's Hospital, where she successively filled the positions of probationary nurse, staff nurse, acting sister, acting night superintendent, and assistant housekeeper, afterwards acting as parish nurse under the Order of St. John of Jerusalem and night superintendent at Chelsea Infirmary.

MANSFIELD ACCIDENT HOSPITAL.—Miss Ransford has been elected Matron of the Accident Hospital at Mansfield. Miss Ransford was trained at St. Mary's Hospital, Paddington, and worked as staff nurse at the Albert Docks Seaman's Hospital, as charge nurse at the East Dulwich Grove Infirmary, and as sister in charge of the Buckhurst Hill Medical Provident Home.

NEW SANATORIUM, HASTINGS.—Miss A. D. Ainsworth has been appointed Matron to this institution. Miss Ainsworth was trained at St. Thomas's Hospital, and has held various posts as nurse and sister, her last appointment being that of matron of the Sanatorium, Cardiff.

### Presentations.

MISS MARGARET TATTAM, assistant to Miss Rachel Frances Lumsden, late hon. superintendent of the Aberdeen Royal Infirmary, and since Miss Lumsden's retirement sister-in-charge at the infirmary, has just been presented on her resignation of this post with gratifying tokens of respect and affection from the nursing and domestic staff. From the sisters and nurses she has received a very handsome travelling bag, and from the night sisters, in addition, a memorandum case with silver fittings. The servants have given a beautiful silver serviette ring with thistle and monogram, and a very handsome copy of Tennyson's poems. There is great regret at Miss Tattam's departure.

### Worcester General Infirmary.

OPENING OF THE NEW NURSES' HOME.

THE picturesque old city of Worcester was looking its very best, in the most genial of spring sunshine, on Tuesday afternoon, when the new nurses' home, just added to the General Infirmary, was formally opened by the Lady Mary Lygon. Half-past three was the hour fixed for the ceremony, and by that time the Rev. W. H. R. Longhurst, the Chairman, Colonel Stallard (secretary), and Miss Herbert, the matron, were welcoming numbers of the invited guests in the open space before the new building. Lady Mary, who came with her popular brother, Earl Beauchamp, arrived punctually, and took up her position on the steps leading to the front door, the sisters and nurses, in groups on either side, looking fresh and neat in their uniforms of dark blue and white-striped linen and blue Oxford shirting. Prayers were said by the Hospital Chaplain, in the unavoidable absence of the Bishop of Worcester, who was unable to keep his intention of being present. Then two small patients—dainty little maidens in pink print frocks and sun bonnets—shyly presented Lady Mary with a handsome bouquet, and after a short speech from Mr. Longhurst, the key was duly turned, the door opened, and, Lady Mary standing aside, the sisters and nurses, followed by Miss Herbert, filed into their new home, ranging themselves on each side of the entrance, while the visitors passed through to the sitting-room opposite. Here a vote of thanks to Lady Mary for her presence was proposed by the Mayor, seconded by Mr. T. Bates, and greeted with cordial cheers, and after a few words from Lord Beauchamp in reply, the guests proceeded to examine and admire the building. Tea was afterwards served in the board-room. Amongst those present were the Dean of Worcester (Dr. Forrest) and Mrs. Forrest, Major Hill, Dr. and Mrs. Walpole-Simmons, Mr. and Mrs. Temple Bourne, Dr. and Mrs. Crowe, Mr. Budd, Mr. Bates, Mr. Gostling, Colonel Norbury, Mr. G. A. Sheppard, Dr. Sweet, Dr. Read, Miss Gordon, Matron of St. Thomas's Hospital, Miss Howes, Matron of the Cheltenham General Hospital, and many others.

The home has been planned and arranged with the greatest care, and the result is admirable. Exteriorly it is in keeping with the hospital, with which it is connected by a covered way, and like the older building is of red brick. On the ground floor are the sitting-rooms for sisters and nurses, divided by folding doors. The windows of these rooms look on to a terrace and garden, where a lawn is to be laid down for tennis as soon as may be, and to which a glass door gives admittance. These rooms are charmingly furnished with warm-looking carpets and plenty of easy chairs cushioned with cheerful cretonne. The walls throughout the home are distempered for the present a soft shade of green, and the tiled fireplaces and mantles are blue-green in tone. The sitting-rooms are hung with good prints, the gift of Mr. Brodie, the father of a former house surgeon. There are seven bedrooms on the ground floor and eleven on each of the other two, the second and the same number above again, while each floor is provided with a good-sized bath-room and a w.c. Box-rooms and a room for bicycles are in the basement. The bedrooms are distinctly larger than the ordinary run of nurses' rooms, and delightfully light and airy, several having two windows, while the ventilation is thorough. The windows are made with the deep lower rail, to allow air to enter between the sashes, and over every door is a fanlight. The sisters' rooms are carpeted with a small square; the probationers' have three mats. The furniture is of polished wood, the dressing-tables and washstands having plenty of drawers and cupboards; some rooms have long cupboards while others have spaces curtained off for hanging dresses. Every room has a fireplace. Some of the floors are painted white, others stained and polished; the hall and ground floor passages are laid with terrazzo. The staircase and entrance hall are wide and airy. The home is lighted throughout with electric light, and a telephone is laid between it and Miss Herbert's room in the hospital.

On Tuesday all the rooms were bright with flowers sent by Lord Beauchamp. Every bedroom is provided with a pretty green pottery vase, the gift from the firm who carried out the furnishing.

The architects of the new home are Messrs. Lewis Sheppard and Son, and the builders Messrs. Wood. In every detail of the building the keenest personal interest has been taken by Mr. Longhurst, and the result is one on which all connected with the Worcester General Infirmary are to be congratulated. The home has been needed for a long time, and it is matter for very great satisfaction that this important forward step in the history of the hospital is now an accomplished fact.

### The Book World for Women and Nurses.

[We invite Correspondence, Criticism, Enquiries, and Notes on Books likely to interest Women and Nurses. Address, Editor, THE HOSPITAL (Nurses' Book World), 28 & 29, Southampton Street, Strand, London, W.C.]

COOKERY FOR INVALIDS AND OTHERS. By LIZZIE HERITAGE. (Published by John Hogg.) 180 pages, price 2s. 6d.

This is an excellent practical little book, which might advantageously be in the possession of every household and every nurse. But, like many such books, it attempts to combine the needs of a nursing clientèle with the ordinary needs of domestic life. The two should invariably be kept separate. The ideal sick cookery book remains to be written, and it can only be satisfactorily written by a trained nurse, for she alone can understand the complexities of invalid dietetics. If Miss Heritage were a nurse she would not state that "arrowroot is found soothing in irritable conditions of stomach and intestines," for this is a long since exploded idea. Neither would she recommend "patent gravy thickeners" and cornflour to be freely used in sick cookery. For, as all nurses know, neither of these has any place in the invalid dietary. But if Miss Heritage is not a nurse, she is plainly an admirable cook, and that is a very excellent thing to be. The book bristles with practical household hints, and her oyster and chicken sandwiches are the most appetising dainties we have come across for a very long time. The fish recipes, too, are most praiseworthy, the more so since fish is not nearly as much used in the sick-room as it should be, partly because so few cooks go beyond cod and oyster sauce or "a nice bit of sole." Now, of these, both good in their way, one soon tires and longs for variety. It is impossible to agree with the author that "savoury and sweet omelettes are well suited to invalids," for the person who can digest the average omelette should certainly not need a nurse in attendance. Neither does boiling "add to the nourishment of milk"; it simply renders it more digestible. These, however, are the natural mistakes which must creep in to the cookery of one who is not at the same time a nurse. Nurses, however, will gain much from this book. For instance, as to the re-warming of beef-tea between two soup plates placed on the top of a saucepan of boiling water. We have seen nurses placing beef-tea into a saucepan not quite scientifically sterilised—to put it delicately—and letting it come to the boil. The book comprises the art of the general cook. And this all nurses have not time to become. For it is quite possible to be a good invalid cook without mastering the whole craft. But Miss Heritage's hints will prove valuable in either case.

### Everybody's Opinion.

[Correspondence on all subjects is invited, but we cannot in any way be responsible for the opinions expressed by our correspondents. No communication can be entertained if the name and address of the correspondent is not given, as a guarantee of good faith but not necessarily for publication, or unless one side of the paper only is written on.]

### ANTISEPTICS FOR NURSES.

"Mr. F. W. Barff" writes: My attention has been called to a statement which occurs in your issue of April 2nd, under "Antiseptics for Nurses," viz., "boroglyceride, which is merely a mixture of boracic acid and glycerine," &c. This is absolutely not the case. Boroglyceride is a true chemical compound of boracic acid and glycerine, patented by my father.

*** We gladly insert the above letter. It is quite clear that all the writer meant was that boroglyceride is compounded entirely of boracic acid and glycerine, which we suppose is the case.—Ed. *T. H.*

### THE USE OF IODINE.

"Inquirer" writes: In the paper on "Antiseptics for Nurses" (for April 2nd), by A Medical Woman, the writer says: "If iodine had not the great disadvantage of staining both the skin and linen brown, it might be far more extensively used." Does your correspondent know that a few drops of carbolic acid (1 in 20) added to the iodine after it is ready prepared for use as a lotion (ʒj ad. Oi.) will turn the lotion white and prevent any staining of skin or linen. I have found the knowledge of this fact very useful myself, and would like to pass it on to others. (2) Will you kindly tell me through "Notes and Queries" the name of a good handbook on the feeding of infants, and the price of the same. Also if there is any good book on diseases of children less bulky and expensive than such works as that of Dr. Eustace Smith.

### INVALID DIET.

"Sister C." writes: An eminent physician desired a patient of mine to take boiled cucumber as a change with other green vegetables, of which, at this time of the year, a variety is rather difficult to get. Not having tasted cucumber boiled, my patient was rather shy of it, but, after having tasted it and finding it so very delicious, we frequently have it as a second vegetable, sometimes boiled in stock, or served like vegetable marrow with melted butter. The flavour of the cucumber boiled is something like asparagus, yet keeping its own individuality, and we find the cost no more than other delicate spring vegetables. I fancy this is not generally known, and I have gained so many kindly hints from The Hospital. There are many private nurses who would be glad to know this, as it is so difficult to get change and surprises in the diet of a convalescent.

### A POINT WORTH NOTING.

"A Matron" writes: Could you put a paragraph in The Hospital drawing nurses' attention to insufficient addresses which are often given when replying to advertisements. I have often received such, and certainly should not try to acknowledge if a stamped envelope had not been enclosed, and after taking a good deal of trouble to find out any name likely to be the one, I have had letters returned "not known here." It not only causes a lot of trouble to those wishful to help them, but must cause much disappointment to themselves, as they naturally are looking each day for some acknowledgement to their application, and I have been very sorry when I have been quite unable to answer. Sometimes the postmark helps one a little, and I try and imitate the name of street and put the name of postmark as the town, hoping the postman might be cleverer than myself, and this has been successful sometimes. I feel really that nurses who are careless in this are very liable to be careless in other matters.

### QUEEN'S NURSES.

"Nurse M. J. L." writes: Would it be trespassing too much on your space to ask if you would insert in your valuable paper a request asking the inspectors and nurses of the Q.V.J.I. for Nurses why a nurse at the end of her engagement should have to return her badge and medal and not be allowed to keep them, or even to be presented with a silver one which she could wear through life. She has nothing to look forward to but her certificates, which she would receive from any institution she worked for in the kingdom. She has risked her life, she has taught the people, and removed very many of the superstitions which they have lived in in the past; and for my own part I have been nearly killed twice in the Atlantic gales during the first six months on Achill Island. Soldiers and others of Her Majesty's subjects have this medal, and why should not the nurses?

*** "Nurse M. J. L." evidently overlooks the distinction which exists between badges and medals. A nurse's badge is worn to mark the fact that she is a member of an association. Naturally when she leaves the association she has no further right to wear a token which refers to membership only. Such badges are not awards in the sense of special medals for special services.—Ed. *T. H.*

### NURSING IN WEST AFRICA.

Miss M. Kingsley writes: Would you kindly allow me to correct a remark that may be misleading? In your issue of April 2nd a nursing sister from West Africa says I gave "the impression that the English had no hospitals out there." I did not do this thing, but referred to those at Sierra Leone and Old Calabar, Lagos and Accra; but I certainly said I had been informed that Sierra Leone, the Gold Coast, and Lagos were in need of further nursing aid. My authorities for this statement were good, and your correspondent's remarks seem to me to bear out their views, for it must have been very hard work for that one nurse at Accra "who had for over four months to work and live alone." But if your correspondent will read the lecture on nursing in West Africa, published practically entire from my notes in this month's *Nursing Journal* I am sure she will see in this matter she has unjustly attacked me; and I sincerely hope she will aid those ladies up here who are deeply interested in attempting to lower the West Coast death-rate, for her knowledge of the Gold Coast hospitals would be of great use. Regarding hospital ships for West Africa, I assure you I do not advocate them from having an old steamer on hand to sell. I leave to professional experts the discussion of the rival claims of ships and shore hospitals. My advocacy of the former in the shape of a hospital cruiser arises from the reasons I have stated—a belief that being out at sea is healthier both for fever patients and nurses. Remember, a good trained nurse is a high-class, valuable form of human being too valuable for West Africa to be allowed to squander them in its wasteful way with life and money. Be it granted that bringing patients out through that Gold Coast surf would now and again lead to a patient getting adrift in it, but the boat boys would pretty certain have him out; but all the boat boys on the Gold Coast cannot get your nurse out of the Gold Coast cemetery she is buried in. I have been through the surf on the Gold Coast, at Accra, and Cape Coast, and I would not speak disrespectfully of anyone's surf, for I know it would hurt my own feelings for anyone to jeer at our South-West coast calema. Nevertheless, you know, merchandise, railway stuff, mine stuff, regiments of soldiers for Ashantee wars, let alone more ordinary people, habitually do survive the Gold Coast surf, and also fever patients, who are "very bad," come out through it now, to try for the chance of life a mail steamer can give them. Surely, such being the case, it is practicable that patients should come through the surf before they are "very bad" for the greater chance a hospital cruiser would give? And I believe I am right in saying that the hospital ship that was on the Gold Coast in connection with the last Ashantee war was found efficient and workable. But if these things are not so, why not build a T-headed pier through the surf? I dared not have mentioned a pier for the Gold Coast two years ago, but as the French have got one now in working order at Kontonon, I venture to make the suggestion.

I apologise for detaining you at such length, but the importance of diminishing the West Coast death-rate is a very great one, and I sincerely hope it will have the co-operation of all the nursing sisters in West Africa brought to bear on it up here in England, even although they may chose to criticise.

### For Reading to the Sick.

"He that loveth not his brother, whom he hath seen, how can he love God whom he hath not seen?"—1 John iv. 10.

**Verses.**

Lovest thou God as thou oughtest, then lovest thou likewise thy brethren.
One is the sun in heaven! and one only, one, is Love also!
Bears not each human figure the God-like stamp on his fore head?
Readest thou not in his face thine origin? Is he not sailing,
Lost like thyself on an ocean unknown, and is he not guided
By the same stars that guide thee?—*Longfellow.*

Since that loving Lord
Commanded us to love them for His sake,
Which in His last bequest He to us spake,
We should them love, and with their needs partake;
Knowing that, whatsoever to them we give,
We give to Him by whom we all do live.—*Spenser.*

Give Me to drink! Above the clouds I dwell.
Sending their rain yet by thy water brink.
Aweary and athirsty I ask for drink
Now as in days of flesh. *Immanuel.*
Give Me to drink; without earth's citadel,
Thirsting I hang upon the bitter tree;
Give Me to drink of my scant water well,
So shall I slake My mighty thirst for thee.

Dost thou not hear My poor about thy portal,
My poor ask drink which cannot stay thirst's pain?
I am the well of Life, the Fount Immortal,
Which whoso drinks shall never thirst again.
And I have said—who hath for mine outpoured
One draught of earth shall lose not his reward.
—*Morgan.*

For all we love, the poor, the sad,
The sinful, unto Thee we call;
O let Thy mercy make us glad;
Thou art our Jesus and our all.
—*F. W. Faber.*

Talk not of wasted affection, affection never was wasted;
If it enrich not the heart of another, its waters returning
Back to their spring, like the rain, shall fill them full of refreshment;
That which the fountain sends forth, returns again to the fountain.
Patience! accomplish thy labour, accomplish thy work of affection!
Sorrow and silence are strong, and patient endurance is godlike,
Purified, strengthened, perfected, and rendered more worthy of heaven! —*Longfellow.*

**Reading.**

We, who are bound to love our neighbours as ourselves, must also pray for them as for ourselves.—*Bishop Jeremy Taylor.*

Kind words are the music of the world. They have a power which seems to be beyond natural causes, as if they were some angels' song which has lost its way, and come on earth. It seems as if they could almost do what in reality God alone can do, soften the hard and angry hearts of men. No one was ever corrected by a sarcasm; crushed, perhaps, if the sarcasm was clever enough, but drawn nearer to God, never.—*F. W. Faber.*

Each solitary kind action that is done the whole world over is working briskly in its own sphere to restore the balance between right and wrong. Kindness has converted more sinners than either zeal, eloquence, or learning; and these three never converted anyone unless they were kind also. The continual sense which a kind heart has of its own need of kindness keeps it humble. Perhaps an act of kindness never dies, but extends the invisible undulation of its influence over the breadth of centuries.—*F. W. Faber.*

### Notes and Queries.

**The contents of the Editor's Letter-box have now reached such unwieldy proportions that it has become necessary to establish a hard and fast rule regarding Answers to Correspondents. In future, all questions requiring replies will continue to be answered in this column without any fee. If an answer is required by letter, a fee of half-a-crown must be enclosed with the note containing the enquiry. We are always pleased to help our numerous correspondents to the fullest extent, and we can trust them to sympathise in the overwhelming amount of writing which makes the new rules a necessity. Every communication must be accompanied by the writer's name and address, otherwise it will receive no attention.**

*District Nurses and Travelling Expenses.*

(28) In accepting an appointment as district nurse I omitted to ask the committee to pay travelling expenses. These they have always paid in previous cases, but as nothing was said about this matter by me before I was engaged they now refuse to pay even part of my travelling expenses. Is this right?—*E. H.*

It is customary for the district nurse's travelling expenses to be paid if she remains more than six months, but you have no redress if it was not agreed upon beforehand.

*How to Become a Nurse.*

(29) What hospital can I enter at 34 years of age to be trained for nursing?—*H.*

Consult Honnor Morten's book, "How to Become a Nurse," published by the Scientific Press, London, price 2s. 6d., where you will find mentioned the hospitals which train nurses at your age.

*Training in Nursing.*

(30) Would you please give me a little information respecting the hospital nurse, as I am intending to take up that profession?—*L. M. (Ireland).*

You will find all particulars as to how you should proceed in Honnor Morten's book, "How to Become a Nurse" (The Scientific Press London, 2s. 6d.)

*Free Probationer.*

(31) I am 29 years of age, and am anxious to become a nurse. Can you advise me as to the best course to adopt? I am anxious to enter a training school as a free probationer.—*L. J. (Peckham).*

See other answers to similar questions. You might address a letter, giving full particulars, to the matrons of large county infirmaries, a list of which you will find in Burdett's "Hospitals and Charities," published by The Scientific Press, London, price 5s.

*Maternity Nursing.*

(32) Could you please tell me of an institute or hospital where I could be trained free or for a small premium as maternity nurse, age 35.—*A Reader of "The Hospital."*

The fees charged by the East-end Mothers' Home, 396, Commercial Road, E., are the lowest.

*Male Nurse.*

(33) I have been acting as valet-attendant for some time, and am anxious to become a fully trained male nurse. What hospitals in London take in male probationers.—*A. H. (Southampton).*

There is no hospital in London or in England that fully trains male nurses. The City Hospital, Blackwell's Island, New York, U.S.A., trains male nurses.

*Home for Incurables.*

(34) Is there a home for incurables where a poor woman could be received free. She has been a cripple from birth.—*Nurse K. M.*

You will find a list of homes for incurables in "Burdett's Hospitals and Charities" (The Scientific Press, London, price 5s.), which also states whether inmates are received free or for payment.

*Convalescent Aid.*

(35) Is there a convalescent home on the South Coast where I could be received free, or, if not, would some sister nurses help me to defray the expense?—*Nurse Agnes.*

Why do you not apply to The Hospital Convalescent Fund, 28, Southampton Street, Strand, the object of which is to give free convalescent aid to poor nurses in need of rest and change?

*Probationer at Thirty-four.*

(36) Is there any limit to the age at which probationers are accepted by the hospitals? My age is 34. Would I be eligible?—*K. M. A., Coventry.*

Very few hospitals would accept a probationer at your age, except as a paying probationer. See answer to (30), above.

*Midwifery Training.*

(37) Will you tell uncertificated midwife how to become one at the smallest expense?—*Anxious.*

See answer to (34), above.

*Nursing on Voyage to Australia.*

(38) I should like to take charge of a patient on the voyage to Australia, or undertake the care of children, in return for free passage. I am a certificated nurse. Could you tell me where to apply?—*Nellie.*

Your best course would be to advertise your wants in the newspapers; also to tell your matron of your desire, as she might have an application for such services.

*Query for Nurses.*

(39) Would any of your correspondents kindly tell me the best way of giving a nutrient enema so as to prevent the air being injected, and what is the best contrivance to use.—*Veritas.*

## Figures and Tables

**Figure f1:**